# Evolution of Cone-Shaped Damage Channels in Aluminum Nanofilm Induced by Repeated High-Power Subpicosecond Terahertz Pulses

**DOI:** 10.3390/nano16120760

**Published:** 2026-06-17

**Authors:** Sergey I. Ashitkov, Oleg V. Chefonov, Andrey V. Ovchinnikov

**Affiliations:** Joint Institute for High Temperatures of the Russian Academy of Sciences (JIHT RAS), Moscow 125412, Russia; ashitkov11@yandex.ru (S.I.A.); oleg.chefonov@gmail.com (O.V.C.)

**Keywords:** terahertz pulses, aluminum nanofilm, local field enhancement, thermomechanical damage

## Abstract

We investigate the formation of surface periodic structures during ablation of a 20 nm aluminum film on a glass substrate by high-power terahertz pulses. Using subpicosecond pulses in the 0.5–3 THz range with a field strength of 15 MV/cm (fluence 0.3 J/cm^2^) generated in a DSTMS crystal pumped by a femtosecond Cr:Forsterite laser, we observe discrete growth of cone-shaped damage channels with a period of 20 µm at an energy density below the single pulse ablation threshold ($F_{a}\approx 0.15$ J/cm^2^). The channel length increases from pulse to pulse (for 8, 20, and 100 pulses) due to local current density enhancement at the channel tip. This enhancement scales inversely with the square root of the tip radius and reaches an order of magnitude. Surface morphology analysis reveals a thermomechanical mechanism governing film destruction. The observed self-organized periodic structures, whose orientation is strictly perpendicular to the THz electric field, hold promise for functional devices in the terahertz band, such as polarizers, near-field sensors, and spatially selective absorbers, provided the formation process can be regulated.

## 1. Introduction

Although the terahertz (THz) range of electromagnetic waves has enormous potential for a wide range of applications in the fields of spectroscopy, visualisation, signal processing and biomedicine [1,2], it is currently much less studied than the microwave, infrared and visible ranges. With the advent of powerful THz radiation sources based on nonlinear crystals with femtosecond optical pumping [3,4] and free-electron lasers (FELs) [5,6], increased interest is shown in their interaction with various materials. This is due to the nonlinear nature of interaction [7,8,9], the specificity of heat transport, surface modification and damage [7,10,11,12,13,14,15,16] during single and multiple exposures to THz pulses.

One of the rapidly developing fields is terahertz plasmonics. Terahertz hybrid plasmonic waveguides based on an elliptical fiber made of a three-dimensional Dirac semimetal are being studied [17,18,19,20], as well as metamaterials for dynamic control of the polarization state of terahertz waves [21] for the development of multifunctional devices in the terahertz range, such as polarizers, waveplates, and modulators. A highly sensitive narrowband terahertz quad-band absorber constructed from bulk Dirac semimetal and silicon dioxide was proposed in [22]. In [23], a multiband absorber using structured graphene was presented. In addition, surface plasmon resonance sensors offer significant advantages in biochemical analysis due to their label-free operation, high sensitivity, and real-time monitoring capability [24].

The use of sub-wavelength structures makes it possible to enhance local electric fields by several orders of magnitude [25]. The acceleration of electrons emitted by a nanotip of a metal needle irradiated with single-cycle THz pulses was described [26]. The observed local field enhancement, inversely proportional to the tip radius, reached several thousand. At the same time, high acceleration efficiency for optical laser pulses has not been achieved due to the high field oscillation frequency.

Recently, single- and multi-pulse ablation of thin metal films on glass substrates irradiated with 20 MV/cm subpicosecond THz pulses focused to a spot at the diffraction limit has been reported [12,13,27]. In contrast to single exposure, the destruction of films caused by multiple THz pulses was observed as regular structures in the form of elongated damage channels or cracks perpendicular to the direction of the electric field and having periods significantly shorter than the incident wavelength. A hypothesis [12] was proposed about the thermomechanical nature of the occurrence of damage initiation centers. However, the mechanism for the development of extended damage channels from pulse to pulse was not explained.

The physics of laser-induced periodic surface structures (LIPSS) formation on various materials exposed to ultrashort laser pulses is still greatly disputed [28,29]. Depending on the period they are divided into two main types. Low-spatial-frequency (LSFLs) LIPSS have a period Λ close to the laser wavelength *λ*, while for high-spatial-frequency (HSFLs) LIPSS Λ≪λ. The mechanism of LSFLs formation is usually based on the interference of incident laser radiation with the scattered surface electromagnetic wave [30]. Formation of HSFLs is less studied [29]. They are commonly described by models of self-organization [31], as well as using an electromagnetic approach, including the near-field scattering effect [32]. In many cases, the HSFL’s period in air is well described by the expression Λ≈(λ/n)/2 (*n* is the refractive index) [28,33].

Several reports on the observation of LIPSS in the THz range have appeared only in the last decade. The detection of fine regular structures similar to HSFL on the surface of silicon wafers after exposure to THz-FEL pulse trains was first reported [5]. As noted, the period of the structures Λ∼λ/25 could not be explained by the mechanisms proposed so far for the optical range. The formation of LIPSS was observed on a Ge_2_Sb_2_Te_5_ sample after exposure to trains of picosecond THz-FEL pulses with an intensity below the ablation threshold [6]. A difference in crystallinity was shown between LIPSS formed on Si irradiated with Ti:Sapphire laser (0.8 µm) and FEL (11.4 µm) [34].

Despite a large number of works, the universal mechanism for LIPSS formation across a wide range of materials and pulse parameters remains unclear [29]. The mechanism for HSFL formation in the THz range cannot be explained by models applicable to optical fs lasers [5]. Studying regular surface structures in the THz range significantly expands the range of available data across the wavelength range.

In this paper, we investigate the dynamics of the formation of regular, extended damage channels in an aluminum nanofilm on a glass substrate exposed to repeated single subpicosecond THz pulses. We propose a model explaining the formation of the subwavelength structure of the damage channel. The observed discrete increase in channel length at intensities below the single-pulse ablation threshold can be explained by a localized enhancement of the field near the channel tip. Unlike exposure to FEL trains, the use of single THz pulses allowed us to establish a correlation between the channel length and the number of pulses.

## 2. Materials and Methods

**Figure 1:**
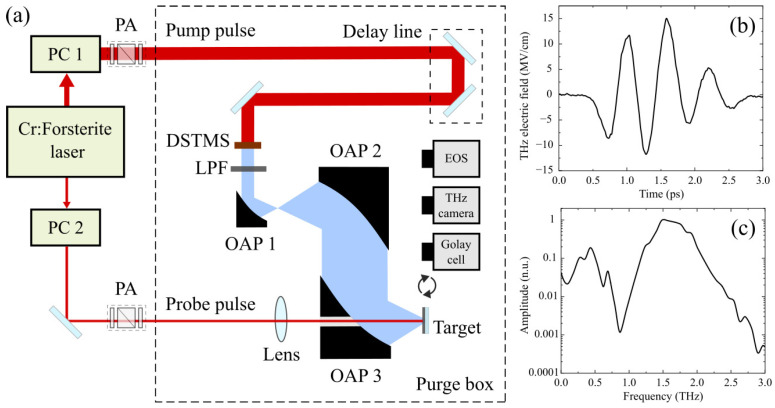
(**a**) Experimental setup. PC 1,2—pulse compressors, PA—polarizing attenuator, LPF—THz low-pass filter, OAP 1,2,3—off-axis parabolic mirrors, EOS—standard electro-optic sampling system consisting of a delay line, a quarter-wave plate, a Wollaston prism, and a balanced detector. (**b**) Temporal profile of the THz electric field. (**c**) Corresponding spectrum.

The experimental setup is shown in Figure 1. Terahertz pulses were generated by optical rectification in a 400-µm-thick organic nonlinear crystal DSTMS [12]. The crystal was pumped by laser pulses of 100 fs duration at a wavelength of 1240 nm, generated by chromium-doped forsterite laser system [35]. A pulse of 700 fs duration was generated in the DSTMS crystal in the 0.5–3 THz range (central wavelength 200 µm). A low-pass THz filter (LPF8.8-47, Tydex, St. Peterburg, Russia) with an optical pump attenuation of at least 10^5^ was used to cut wavelengths shorter than 34 µm. To compensate for the terahertz beam divergence, a 6:1 telescope was used, consisting of two off-axis parabolic mirrors with focal lengths of 25.4 and 152.4 mm. The terahertz beam was focused normal to the target into a diffraction-limited spot using an off-axis parabolic mirror with a focal length of 50.8 mm and a diameter of 50.8 mm. Experiments were performed with a 20-nanometer-thick aluminum film deposited by magnetron sputtering on a polished glass substrate 160 µm thick. The terahertz pulse energy was measured with a calibrated optoacoustic detector (Golay cell, GC-1D Tydex, St. Peterburg, Russia). The maximum pulse energy was equal to 80 µJ. The polarized attenuator placed in the pump laser beam was used to adjust the THz pulse energy. A more detailed description of the experimental setup is given in [12,13,27].

**Figure 2:**
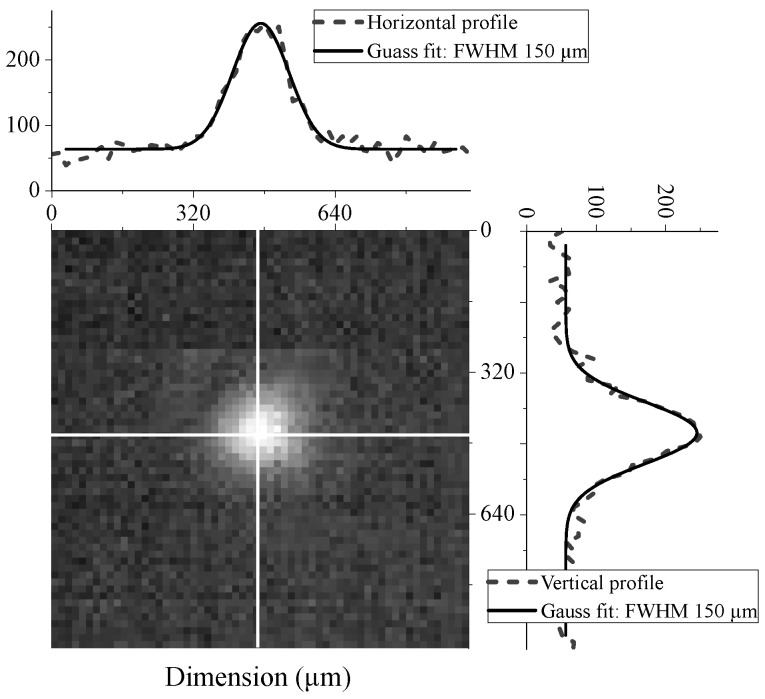
THz beam profile in the beam waist for the DSTMS crystal.

The ablation threshold $F_{a}$ was determined by the well-known technique [36] for beams with a Gaussian spatial distribution by measuring the dependence of the radius *r* of the damage (hole in the film) on the pulse energy. The measured values of the beam radius at the $e^{-1}$ level and the single-shot ablation threshold, respectively, were $r_{0}\approx 90$ µm and $F_{a}\approx 0.15$ J/cm^2^. Figure 2 shows the spatial distribution of the THz beam in the focal plane of OAP 3 recorded by the THz camera, together with the corresponding cross-sectional profiles. The beam profiles at the focus are symmetric and well approximated by a Gaussian distribution. The spot is circular, with a full width at half maximum (FWHM) of ≈150 µm, which is in good agreement with the measurements ($r_{e^{-1}}=FWHM/\sqrt{4\ln2}$).

## 3. Results

**Figure 3:**
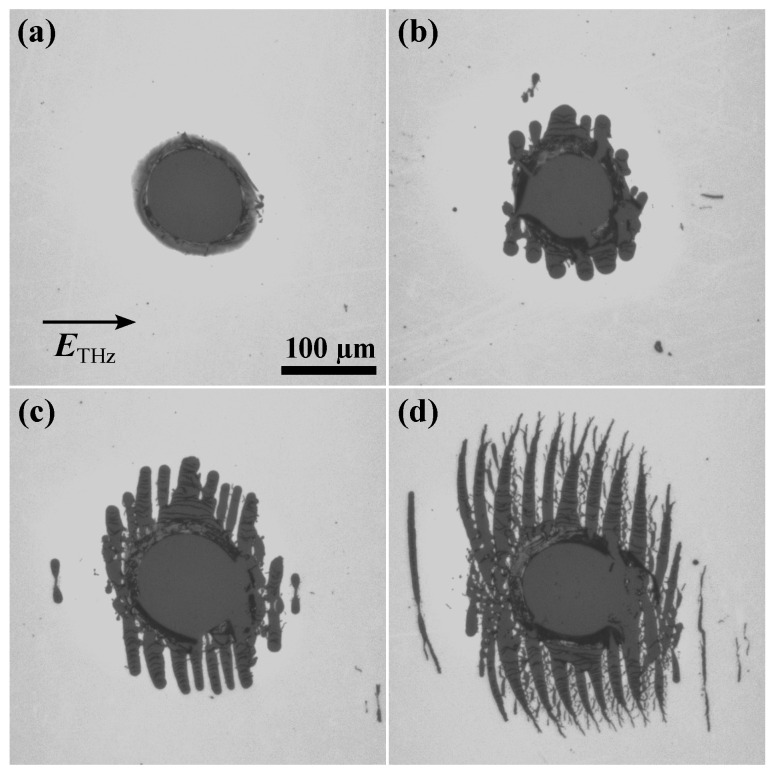
Evolution of damage in a 20 nm thick aluminum film after exposure to THz pulses with a fluence of 0.3 J/cm^2^: (**a**) 1 pulse, (**b**) 8 pulses, (**c**) 20 pulses, (**d**) 100 pulses. The arrow indicates the direction of the THz electric field. The scale bar in (**a**) applies to all panels.

Figure 3 shows images of the aluminum film damage after single and multiple exposures to THz pulses with an energy density of $F_{0}=0.3$ J/cm^2^ (electric field strength of 15 MV/cm), obtained using an optical Olympus inverted microscope with PLN 10× objective.

After the first THz pulse, a through circular hole forms in the film (Figure 3a). After irradiation by several pulses, the damage develops into a spatially periodic structure consisting of elongated cone-shaped channels oriented perpendicular to the THz field polarization (Figure 3b–d).

**Figure 4:**
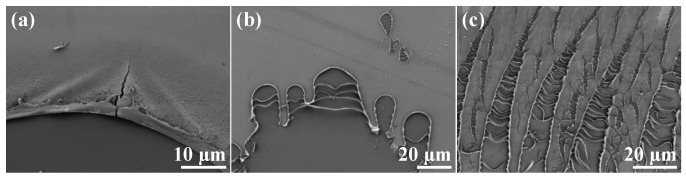
SEM images of the damaged area on the aluminum film after exposure to (**a**) 1, (**b**) 8, and (**c**) 100 THz pulses.

Figure 4 shows enlarged images of the damage fragments, obtained by scanning electron microscope (SEM) after single and multiple THz pulses with $F_{0}=0.3$ J/cm^2^.

After a single THz pulse at $F_{0}=0.3$ J/cm^2^, the aluminum film in the center of the spot is completely removed from the glass surface (Figure 4a). A distinctive feature is the presence of a rim approximately 1–2 µm wide along the fracture boundary, representing the rolled edge of the damaged film. A modified region is also observed outside the damage boundary, where the film surface exhibits greater roughness compared to the original one. The structure of the modified surface convincingly indicates film melting. The presence of a crack and delamination also indicates the action of mechanical stress. The threshold energy density corresponding to this area was approximately $F_{mod}\approx 0.5F_{a}$.

The measured period of the structures is 20 ± 2 µm (based on the analysis of several channels in Figure 3d and Figure 4c). After exposure to 100 pulses, the length of the longest channels reaches ~150 µm from the spot center. According to the Gaussian distribution, the ratio of the electric field strength at the center and at this distance is 11. The channel width near the spot center is ~20 µm, while at the periphery the channel narrows to ~1 µm. In the experiments, the repetition rate of the 700 fs THz pulses was 10 Hz.

## 4. Discussion

Let us estimate the temperature and thermomechanical stress in the aluminum film. The specific properties of thin metal films in the THz range with a thickness much smaller than the skin layer are characterized by high reflection and absorption. They have a maximum absorption of up to 50% at a critical thickness (ranging from units to tens of nanometers), which depends on the conductivity [37,38]. In the THz range, in case of the normal skin effect, the reflection *R*, transmission *T* and absorption *A* coefficients at normal incidence can be found from the relations [37]:

(1) $$R=\beta^{2}/{\left(1+\beta \right)}^{2};\;T=1/{\left(1+\beta \right)}^{2};\;A=1-R-T.$$

Here β=2πσd/c, where *c* is the speed of light. As can be seen in the case of the normal skin effect, the parameter *β* is independent of wavelength, but is determined by the film thickness *d* and conductivity *σ*. According to [39], the conductivity of an aluminum nanofilm in the THz range is more than two times less than the conductivity of bulk [40] which is equal to $4\times 10^{17}$ s^−1^ (Gaussian system). Assuming for an aluminum nanofilm $\sigma \approx 10^{17}$ s^−1^ from relations (1), we obtain R=0.95, $T=5\times 10^{-3}$, A=0.045.

Irradiation of a metal with an ultrashort THz pulse results in nonequilibrium heating of electrons [11], whose temperature during the two-temperature stage significantly exceeds the lattice temperature. For aluminum, the characteristic lattice heating time is 1–2 ps. For ultrathin films, neglecting thermal conductivity simplifies the solution and allows one to estimate the lattice temperature, which determines the magnitude of the thermoelastic stress. In the case of complete melting of a film, the temperature *T* and melting threshold $F_{m}$ can be found from the expression [41]:

(2) $$\frac{AF}{d}=\rho_{0}\left[C_{l}\left(T-T_{0}\right)+\Delta H_{m}\right],$$

where the density of aluminum is $\rho_{0}=2.7$ g/cm^3^, the specific heat capacity $C_{l}$ and enthalpy of fusion $\Delta H_{m}$ are 0.88 J/(g·K), $3.9\times 10^{2}$ J/g, respectively [40], $T_{0}=293$ K is the initial temperature of the sample. For the aluminum melting point T=933 K, the estimated melting threshold value $F_{m}=0.11$ J/cm^2^ according to (2) is in good agreement with the modification threshold $F_{mod}$ determined above and, along with the surface morphology, indicates the presence of film melting. The temperatures at the center of the spot at $F_{0}$ and near the ablation threshold $F_{a}$ were 2640 and 1280 K, respectively.

To evaluate the mechanical stress generated in the Al film under ultrafast heating, the approximate expression may be used [42]:

(3) $$\sigma_{stress}=\frac{E_{elast}\alpha \left(T-T_{0}\right)}{2\left(1-\mu \right)},$$

where for aluminum $E_{elast}=70$ GPa is the Young’s modulus, $\alpha =32\times 10^{-6}$ K^−1^ is the coefficient of linear thermal expansion, μ=0.31 is the Poisson ratio [40], and $\left(T-T_{0}\right)$ is the temperature difference along the radius of the spot. Assuming T=900 K, we obtain an estimate of the thermomechanical stress $\sigma_{stress}\approx 1$ GPa when heating the film to the melting point. This value is an order of magnitude greater than the characteristic adhesion of metal films [43] and is also comparable to the dynamic yield strength of aluminum in the picosecond loading range [44]. Consequently, at temperatures below the melting point, delamination and cracking of the film may occur. Cracking can serve as a precursor for further damage with repeated irradiation [12].

Stress relaxation occurs by expanding the film in the direction normal to the substrate. Reflection of the rarefaction wave from the rigid substrate leads to a concentration of tensile stress at the film/substrate interface and separation of the film from the substrate [45]. Thus, due to rapid melting and thermomechanical expansion, the film is removed in the center of the irradiated area, forming a circular roll along its boundary.

According to [46] the absorption length of THz radiation in glass, depending on the frequency in the range from 0.5 to 10 THz, can vary from 500 to 10 µm. For the spectrum of our pulse the measured absorption length in glass was approximately 80 µm. Considering the low transmittance of Al film, it can be assumed that the volumetric absorbed energy density of THz radiation in the glass substrate and, accordingly, its heating will be extremely small compared to the heating of the metal film. After the film is removed in the central region, subsequent THz pulses pass through the glass. Calculations show that the substrate heating does not exceed 25 K and does not affect the observed effects.

A study of the surface morphology revealed that, upon repeated exposure to THz pulses, damage (complete film removal) forms as a spatially periodic structure of extended cone-shaped channels oriented perpendicular to the polarization of the THz radiation (Figure 3b–d). As the number of pulses *N* increases, the channel length increases discretely with *N*, and the channel width decreases. The maximum channel width near the central area reaches approximately 20 µm and decreases to submicron dimensions at the periphery (Figure 4c). It is important to note that with repeated exposure, damage occurs in the region where the energy density is below the single-shot ablation threshold $F_{a}$.

**Figure 5:**
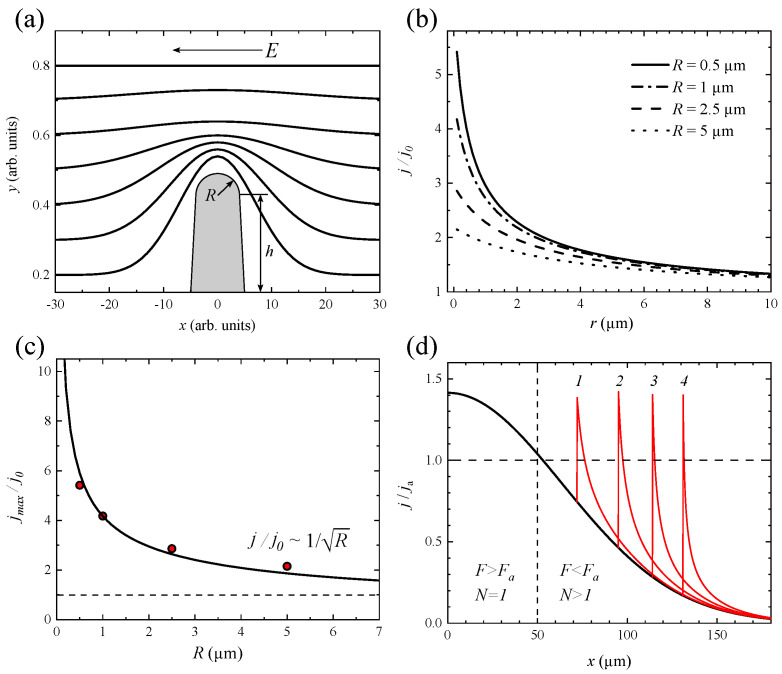
(**a**) illustration of the local current enhancement near the tip of the damage channel; (**b**) normalized radial distribution of the current near the tip of the damage for different *R* at a fixed length of *h* = 20 µm; (**c**) dependence of the current amplification $j_{max}/j_{0}$ on the radius *R* of the tip (maximum amplification values from (**b**) (red dots); line is the fitting curve); (**d**) local current density enhancement at the tip of the damage channel with a radius *R* below the ablation threshold: 1—8 µm, 2—5 µm, 3—2.5 µm, 4—1 µm. $j_{a}$ is the current density corresponding to the ablation threshold. Gaussian distribution of THz field is plotted with a solid black line.

The THz field penetrates the entire film, inducing a current. The current density is determined by Ohm’s law $\vec{j}=\sigma \vec{E}$ (where $\vec{E}$ is the electric field and *σ* is the conductivity). The mechanism of damage during repeated exposure may be related to a local current enhancement near the channel tip (Figure 5a). As a result, the micro region near the channel tip is locally heated and destroyed.

The radial distribution of the current density j(r) (Figure 5b) near the tip of the damage channel with radius *R* can be described within the quasi-static approximation by the expression [47]:

(4) $$j\left(r\right)=j_{0}\frac{r-R/2+h}{\sqrt{{\left(r-R/2\right)}^{2}+2\left(r-R/2\right)h}}$$

where $j_{0}$ is the initial current density far from the damage, *h* is the channel length, and *r* is the distance from the channel tip. It should be noted that the current density calculation within the quasi-static approximation is also valid for a THz pulse, since the characteristic channel dimensions are smaller than the THz wavelength.

Figure 5b shows that near the tip, the current density is several times higher than far from the damage zone. The characteristic size of the current enhancement (and, consequently, the damage area) is comparable to the radius *R* of the tip. After each subsequent pulse, the channel length increases by approximately this amount, and the channel grows in a direction perpendicular to the electric field $\vec{E}$.

From expression (4) it follows that the maximum value of the local current density amplification is inversely proportional to the square root of the channel tip radius $j_{max}/j_{0}∼1/\sqrt{R}$ (the line in Figure 5c). It also follows that in the case of exposure to a THz pulse with a Gaussian intensity distribution, the width of the damage channel should decrease with increasing distance from the center of the impact area, where the field strength is lower. This is illustrated in Figure 5d. The area marked as $F>F_{a}$ in Figure 5d corresponds to the ablation zone after exposure to the first pulse. For subsequent pulses, to create the current density necessary for destruction, the radius of the damage channel tip should decrease with increasing *N*. Thus, at a distance of 130 µm from the center of the impact area, the radius of the channel tip should be equal to 1 µm (curve 4 in Figure 5d). The corresponding magnitude of the local field amplification at the tip of the channel exceeds by a factor of 8. After each localized damage of the film, a rim of solidified melt forms along its boundary. The thickness of the rim is greater than that of the film. Therefore, upon a subsequent THz pulse, a rim usually does not fail, but rather persists as a delaminated bridge (Figure 4b,c).

**Figure 6:**
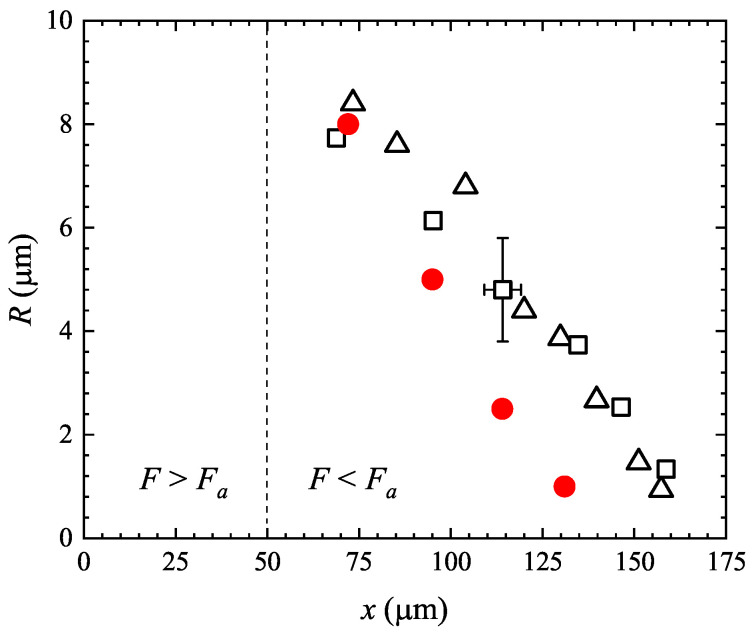
Dependence of the radius of the tip of the damage channel under multi-pulse irradiation on the distance from the center of the focal spot. Squares and triangles are experimental data; Red circles are the calculation result.

Figure 6 demonstrates a comparison of the calculated channel radius *R* as a function of distance from the spot center *x* with the experimental data.

In Figure 6, the empty symbols show the result of processing two damage channels near the equatorial part of the structure in Figure 3d. Here, the radius of the channel tip *R* at a distance *x* is taken to be half its width. The red circles show the calculation results from Figure 5d. The vertical dotted line corresponds to the radius of the central spot (the boundary of the single-pulse ablation region). Figure 6 demonstrates good agreement between the experimental and calculated data. The observed discrepancy at a distance greater than 90 µm can be explained by the beam’s deviation from the Gaussian distribution below the $1/e^{2}$ level.

The spatial periodicity of the channels may be related to local field enhancement induced by inhomogeneities. Defects arising under the influence of thermomechanical stresses after irradiation with one or more pulses may serve as seeds for the development of fracture channels [12]. The observed period between channels is approximately 20 µm.

In the optical range, the spatial period of HSFLs is usually described by the expression Λ=λ/(2n), where *n* is the refractive index of the medium, which generally depends on the intensity [28,33]. In the case under consideration, this expression does not directly describe the periodicity of damage channels due to the large value of *n* for metals in the THz range (for Al at a wavelength of 200 µm, n≈400 [48]). The expected period of HSFLs in this case yields a value two orders of magnitude smaller than that observed experimentally. Such a discrepancy may be due to the need to take into account a number of factors, such as a nonlinearity of the refractive index [49] and environmental factors [50]. A nanoscale metal film should probably be considered together with the dielectric substrate. In the THz range under consideration, the value of the real part of the refractive index of glass is 2.25 [50]. This yields a magnitude of the estimated spatial period of the structure, Λ∼40 µm, closer to the experimentally observed value.

## 5. Conclusions

In summary, we investigated the fracture dynamics of a 20-nanometer-thick aluminum film on a glass substrate subjected to repeated irradiation to single THz pulses with a Gaussian field distribution (duration 0.7 ps, central wavelength 200 µm, field strength 15 MV/cm). Below the single-shot ablation threshold, spatially periodic structures arise on the surface in the form of extended, cone-shaped damage channels oriented perpendicular to the electric field direction. The damage channel length increases discretely, proportional to the number of applied THz pulses. A model for the development of damage channels is proposed, based on the local field enhancement at the channel tip. The magnitude of amplification is inversely proportional to the square root of the tip radius. The presented estimates and the surface morphology indicate a thermomechanical fracture mechanism. The observed periodicity of the damage channels is not described by LIPSS formation models accepted for the optical range.

## Data Availability

Data will be made available on request.

## References

[1] Mittleman D.M. (2017). Perspective: Terahertz science and technology. J. Appl. Phys..

[2] Jin Z., Lou J., Shu F., Hong Z., Qiu C.W. (2025). Advances in Nanoengineered Terahertz Technology: Generation, Modulation, and Bio-Applications. Research.

[3] Vicario C., Ovchinnikov A.V., Ashitkov S.I., Agranat M.B., Fortov V.E., Hauri C.P. (2014). Generation of 09-mJ THz pulses in DSTMS pumped by a Cr:Mg_2_SiO_4_ laser. Opt. Lett..

[4] Kim H., Kang C., Jang D., Roh Y., Lee S.H., Lee J.W., Sung J.H., Lee S.K., Kim K.Y. (2024). Ionizing terahertz waves with 260 MV/cm from scalable optical rectification. Light. Sci. Appl..

[5] Irizawa A., Suga S., Nagashima T., Higashiya A., Hashida M., Sakabe S. (2017). Laser-induced fine structures on silicon exposed to THz-FEL. Appl. Phys. Lett..

[6] Makino K., Kato K., Takano K., Saito Y., Tominaga J., Nakano T., Isoyama G., Nakajima M. (2018). Significant Volume Expansion as a Precursor to Ablation and Micropattern Formation in Phase Change Material Induced by Intense Terahertz Pulses. Sci. Rep..

[7] Chefonov O.V., Ovchinnikov A.V., Agranat M.B., Fortov V.E., Efimenko E.S., Stepanov A.N., Savel’ev A.B. (2018). Nonlinear transfer of an intense few-cycle terahertz pulse through opaque n-doped Si. Phys. Rev. B.

[8] Chefonov O.V., Ovchinnikov A.V., Sitnikov D.S., Agranat M.B. (2019). Focal Spot Imaging of Terahertz Subpicosecond Pulse by THz-Field-Induced Optical Second Harmonic Generation. High Temp..

[9] Chefonov O.V., Ovchinnikov A.V., Agranat M.B. (2023). Generation of the Second Optical Harmonic under the Action of Narrowband Terahertz Pulses in the Antiferromagnet NiO. High Temp..

[10] Shalaby M., Vicario C., Hauri C.P. (2016). Low frequency terahertz-induced demagnetization in ferromagnetic nickel. Appl. Phys. Lett..

[11] Bezhanov S.G., Uryupin S.A. (2018). Nonlinear transmission and reflection of a strong terahertz pulse by a metal film. Opt. Lett..

[12] Agranat M., Chefonov O., Ovchinnikov A., Ashitkov S., Fortov V., Kondratenko P. (2018). Damage in a Thin Metal Film by High-Power Terahertz Radiation. Phys. Rev. Lett..

[13] Chefonov O.V., Ovchinnikov A., Evlashin S.A., Agranat M.B. (2019). Degradation and Destruction of Thin Steel Films under Repeated Exposure to Ultrashort Pulses of THz Radiation. Tech. Phys. Lett..

[14] Makin V.S., Makin R.S. (2020). The universal character of the breakdown of condensed media by powerful terahertz radiation and the Abbe criterion. J. Opt. Technol..

[15] Neeraj K., Sharma A., Almeida M., Matthes P., Samad F., Salvan G., Hellwig O., Bonetti S. (2022). Terahertz charge and spin transport in metallic ferromagnets: The role of crystalline and magnetic order. Appl. Phys. Lett..

[16] Ashitkov S.I., Komarov P.S., Ovchinnikov A.V., Romashevskiy S.A., Struleva E.V., Chefonov O.V., Agranat M.B. (2024). Nonequilibrium Heating of Electrons, Melting, and Modification of a Nickel Nanofilm by an Ultrashort Terahertz Pulse. JETP Lett..

[17] Wang G., Cao W., He X. (2023). 3D Dirac Semimetal Elliptical Fiber Supported THz Tunable Hybrid Plasmonic Waveguides. IEEE J. Sel. Top. Quantum Electron..

[18] He X., Cao W., Lin F. (2025). Tunable BIC metamaterials with Dirac semimetals. Nanophotonics.

[19] Cheng Y., Cao W., He X. (2024). Hybrid Plasmonic Waveguides with Tunable ENZ Phenomenon Supported by 3D Dirac Semimetals. Laser Photonics Rev..

[20] He X., Lin F., Liu F., Shi W. (2021). Tunable terahertz Dirac-semimetal hybrid plasmonic waveguides. Opt. Mater. Express.

[21] Liu S., Cao W., Li J., He X. (2025). 3D Dirac Semimetal Metamaterial Supported Terahertz Tunable Dual-Functional Polarization Converter. IEEE J. Sel. Top. Quantum Electron..

[22] Fu S., Yang X. (2026). A terahertz four-band high-sensitivity perfect absorber based on Dirac semimetal. Phys. Lett. A.

[23] Zeng N., Chen Z., Yi Z., Cheng S., Ahmad S., Tang C., Gao F., Li B. (2026). Terahertz multi-band tunable refractive index sensing graphene absorber based on surface plasmon resonance. Phys. B Condens. Matter.

[24] Li Y., Liu W., Liu R., Gao J., Feng J., Xu S., Li Z., Jiang S., Du X. (2023). 3D hybrid arrayed Ag/MOF multi-plasmon resonant cavity system for high-performance SPR sensing. Opt. Laser Technol..

[25] Bahk Y.M., Park D.J., Kim D.S. (2019). Terahertz field confinement and enhancement in various sub-wavelength structures. J. Appl. Phys..

[26] Li S., Jones R.R. (2016). High-energy electron emission from metallic nano-tips driven by intense single-cycle terahertz pulses. Nat. Commun..

[27] Chefonov O., Ovchinnikov A., Evlashin S., Agranat M. (2018). Damage Threshold of Ni Thin Film by Terahertz Pulses. J. Infrared Millim. Terahertz Waves.

[28] Buividas R., Mikutis M., Juodkazis S. (2014). Surface and bulk structuring of materials by ripples with long and short laser pulses: Recent advances. Prog. Quantum Electron..

[29] Bonse J., Gräf S. (2021). Ten Open Questions about Laser-Induced Periodic Surface Structures. Nanomaterials.

[30] Sipe J.E., Young J.F., Preston J.S., van Driel H.M. (1983). Laser-induced periodic surface structure. I. Theory. Phys. Rev. B.

[31] Reif J., Varlamova O., Uhlig S., Varlamov S., Bestehorn M. (2014). On the physics of self-organized nanostructure formation upon femtosecond laser ablation. Appl. Phys. A.

[32] Rudenko A., Colombier J.P., Höhm S., Rosenfeld A., Krüger J., Bonse J., Itina T.E. (2017). Spontaneous periodic ordering on the surface and in the bulk of dielectrics irradiated by ultrafast laser: A shared electromagnetic origin. Sci. Rep..

[33] Liang F., Vallée R., Chin S.L. (2012). Mechanism of nanograting formation on the surface of fused silica. Opt. Express.

[34] Miyagawa R., Kamibayashi D., Nakamura H., Hashida M., Zen H., Somekawa T., Matsuoka T., Ogura H., Sagae D., Seto Y. (2022). Crystallinity in periodic nanostructure surface on Si substrates induced by near- and mid-infrared femtosecond laser irradiation. Sci. Rep..

[35] Agranat M.B., Ashitkov S.I., Ivanov A.A., Konyashchenko A.V., Ovchinnikov A.V., Fortov V.E. (2004). Terawatt femtosecond Cr: Forsterite laser system. Quantum Electron..

[36] Liu J.M. (1982). Simple technique for measurements of pulsed Gaussian-beam spot sizes. Opt. Lett..

[37] Fahy S., Kittel C., Louie S.G. (1988). Electromagnetic screening by metals. Am. J. Phys..

[38] Hadni A., Gerbaux X. (1990). Infrared and millimeter wave absorber structures for thermal detectors. Infrared Phys..

[39] Laman N., Grischkowsky D. (2008). Terahertz conductivity of thin metal films. Appl. Phys. Lett..

[40] Grigoriev I.S., Meilikhov E.Z., Radzig A.A. (1997). Handbook of Physical Quantities.

[41] Viertel T., Pabst L., Ebert R., Exner H. (2019). Selective rear-side ablation of aluminum thin layers with ultrashort-pulsed laser radiation. Appl. Phys. A.

[42] Meshcheryakov Y.P., Shugaev M.V., Mattle T., Lippert T., Bulgakova N.M. (2013). Role of thermal stresses on pulsed laser irradiation of thin films under conditions of microbump formation and nonvaporization forward transfer. Appl. Phys. A.

[43] Vianco P., Sifford C., Romero J. (1997). Resistivity and adhesive strength of thin film metallizations on single crystal quartz. IEEE Trans. Ultrason. Ferroelectr. Freq. Control.

[44] Ashitkov S.I., Agranat M.B., Kanel’ G.I., Komarov P.S., Fortov V.E. (2010). Behavior of aluminum near an ultimate theoretical strength in experiments with femtosecond laser pulses. JETP Lett..

[45] Ivanov D.S., Rethfeld B., O’Connor G.M., Glynn T.J., Volkov A.N., Zhigilei L.V. (2008). The mechanism of nanobump formation in femtosecond pulse laser nanostructuring of thin metal films. Appl. Phys. A.

[46] Tostanoski N.J., Sundaram S.K. (2023). Universal power-law of terahertz optical properties of borosilicate, tellurite, and chalcogenide glass families. Sci. Rep..

[47] Emel’yanov O.A. (2008). Local fracture of thin metallic films during electromagnetic loading. Tech. Phys..

[48] Rakić A.D. (1995). Algorithm for the determination of intrinsic optical constants of metal films: Application to aluminum. Appl. Opt..

[49] Chefonov O., Evlashin S., Ovchinnikova M., Il’ina I., Ovchinnikov A. (2026). Optical properties of thin nickel film exposed to high-intensity terahertz pulses. Tech. Phys. Lett..

[50] Wang C., Huo H., Johnson M., Shen M., Mazur E. (2010). The thresholds of surface nano-/micro-morphology modifications with femtosecond laser pulse irradiations. Nanotechnology.

